# Food for Thought

**Published:** 1895-05

**Authors:** 


### Food for Thought.

How was it that Marion Sims, Flint, Agnew, Keating, Fordyce Barker, Sir Andrew Clark, Charcot, Billroth, and others, had so much time for literary work? And yet their professional duties were certainly as pressing as any one's we know. It would seem that they felt the necessity of keeping their brains in good working order by writing; and if they thought so, no one could hardly be excused from saying, "Oh! I can find no time for writing."—*Rhode Island Medical Science Monthly.*

